# Microcystin-leucine arginine induces the proliferation of cholangiocytes and cholangiocarcinoma cells through the activation of the Wnt/β-catenin signaling pathway

**DOI:** 10.1016/j.heliyon.2024.e30104

**Published:** 2024-04-26

**Authors:** Suppakrit Kongsintaweesuk, Sirinapha Klungsaeng, Kitti Intuyod, Anchalee Techasen, Chawalit Pairojkul, Vor Luvira, Somchai Pinlaor, Porntip Pinlaor

**Affiliations:** a Centre for Research and Development of Medical Diagnostic Laboratories, Faculty of Associated Medical Sciences, Khon Kaen University, Khon Kaen 40002, Thailand; b Medical Sciences Program, Faculty of Associated Medical Sciences, Khon Kaen University, Khon Kaen 40002, Thailand; c Department of Parasitology, Faculty of Medicine, Khon Kaen University, Khon Kaen 40002, Thailand; d Department of Pathology, Faculty of Medicine, Khon Kaen University, Khon Kaen 40002, Thailand; e School of Medical Technology, Faculty of Associated Medical Sciences, Khon Kaen University, Khon Kaen 40002, Thailand; f Department of Surgery, Faculty of Medicine, Khon Kaen University, Khon Kaen 40002, Thailand; g Cholangiocarcinoma Research Institute, Khon Kaen University, Khon Kaen 40002, Thailand

**Keywords:** Carcinogenesis, Cyanotoxin, Cyanobacteria, Environmental exposure, Bile duct cancer

## Abstract

**Background:**

Microcystin-leucine arginine (MC-LR) is a cyanobacterial hepatotoxic toxin found in water sources worldwide, including in northeastern Thailand, where opisthorchiasis-associated cholangiocarcinoma (CCA) is most prevalent. MC-LR is a potential carcinogen; however, its involvement in liver fluke-associated CCA remains ambiguous. Here, we aimed to evaluate the effect of MC-LR on the progression of CCA via the Wnt/β-catenin pathway *in vitro*.

**Methods:**

Cell division, migration, cell cycle transition, and MC-LR transporter expression were evaluated *in vitro* through MTT assay, wound healing assay, flow cytometry, and immunofluorescence staining, respectively. Following a 24-h treatment of cultured cells with 1, 10, 100, and 1,000 nM of MC-LR, the proliferative effect of MC-LR on the Wnt/β-catenin signaling pathway was investigated using immunoblotting and qRT-PCR analysis. Immunohistochemistry was used to determine β-catenin expression in CCA tissue compared to adjacent tissue.

**Results:**

Human immortalized cholangiocyte cells (MMNK-1) and a human cell line established from opisthorchiasis-associated CCA (KKU-213B) expressed the MC-LR transporter and internalized MC-LR. Exposure to 10 nM and 100 nM of MC-LR notably enhanced cells division and migration in both cell lines (P < 0.05) and markedly elevated the percentage of S phase cells (P < 0.05). MC-LR elevated PP2A expression by activating the Wnt/β-catenin signaling pathway and suppressing phosphatase activity. Inhibition of the β-catenin destruction complex genes (*Axin1* and *APC*) led to the upregulation of β-catenin and its downstream target genes (*Cyclin D1* and *c-Jun*). Inhibition of Wnt/β-catenin signaling by MSAB confirmed these results. Additionally, β-catenin was significantly expressed in cancerous tissue compared to adjacent areas (P < 0.001).

**Conclusions:**

Our findings suggest that MC-LR promotes cell proliferation and progression of CCA through Wnt/β-catenin pathway. Further evaluation using *invivo* experiments is needed to confirm this observation. This finding could promote health awareness regarding MC-LR intake and risk of CCA.

## Highlights

• Cholangiocytes and CCA cells internalize MC-LR, leading to increased proliferation.
• MC-LR initiates the cell cycle transition from G0/G1 to S phase.
• MC-LR induces cell division via Wnt/β-catenin signaling pathway.
• β-catenin was significantly upregulated in cancerous tissue of CCA patients.

## Abbreviations

APC: Adenomatous polyposis coli
β–actin: Beta-actin
β–catenin: Beta-catenin
CCA: Cholangiocarcinoma
*c*-*Jun*: Transcription factor Jun
DMSO: Dimethyl sulfoxide
MCs: Microcystins
MC-LR: Microcystin-leucine arginine
MSAB: Methyl 3-[[(4-methylphenyl) sulfonyl] amino] benzoate
OATP1B3: Organic anion transporting polypeptide 1B3
PP2A: Protein phosphatase 2A
*TCF*: T cell factor

## 1 Introduction

In recent times, the risk of water pollution has increased due to the dramatic growth in human activity. The discharge of chemical waste has led to overgrowth of cyanobacteria and the occurrence of cyanotoxins in water reservoirs. MC-LR, a type of cyanotoxin, is widely spread and recognized as a hepatotoxin, being found e.g., in China, the United States, Mozambique [[1], [2], [3]], and also in northeast Thailand [4]. Numerous studies have suggested connections between liver disease and microcystin exposure [[5], [6], [7]]. The consumption of water polluted with MC-LR has been linked to potential carcinogenic effects [8,9]. However, limited research has been performed on the effects of exposure to MCs on the bile-duct cancer development and progression.

A malignant neoplasm that develops from the bile-duct epithelial cells is called cholangiocarcinoma (CCA). This cancer imposes a significant health burden, particularly in East Asian countries, including the Greater Mekong subregion [10]. The primary etiology of CCA in this region is thought to be infection with *Opisthorchis viverrini*, the liver fluke [11,12], although the exact etiological factors responsible for its development are incompletely understood. Exogenous factors such as nitrosamine and other carcinogens could also participate in promoting CCA [13]. Hence, it is not clear whether exposure to a potential carcinogen, such as MC-LR, has the potential to promote or enhance progression of CCA.

Various signaling pathways contribute to cholangiocarcinogenesis [14,15]. Among these, the Wnt/β-catenin signaling pathway plays a critical role in the migration, metastasis, and proliferation of malignant cells [16]. Interestingly, in regions of the world where liver fluke infection is endemic, a link between Wnt signaling pathway and CCA has been identified [17]. The β-catenin destruction complex closely regulates the cellular levels of β-catenin, which plays a critical role in the canonical Wnt/β-catenin pathway. MC-LR primarily targets and inhibits protein phosphatase 2A (PP2A). Prior research has suggested that PP2A has a significant role in regulating the β-catenin destruction complex [18]. The disruption of Wnt/β-catenin pathway is linked to the progression of cancer in different types of tumors [[19], [20], [21]]. A worse prognosis for CCA patients and the occurrence of multidrug resistance are related to increased β-catenin expression [22]. A previous report has shown that MC-LR could promote colon cancer growth through Wnt/β-catenin signaling pathway [23].

Treatment option for CCA varies depending on the staging of disease. However, surgical removal remains the sole curative treatment for early-stage CCA, significantly enhancing survival rates. Unfortunately, in northeastern Thailand, delayed diagnoses often result in patients presenting at an advanced stage, hindering timely surgical interventions [24,25]. Recent studies have revealed a close relationship between the onset and progression of several cancer types and dysregulation in the Wnt/β-catenin signaling pathway [19,26,27]. Thus, many therapies have been designed to target this pathway [28]. However, there is a paucity of research investigating the specific role of MC-LR in relation to the Wnt/β-catenin signaling pathway and its impact on CCA development.

Our goal in this study is to examine the potential impact of MC-LR on the development and progression of CCA via modulation the Wnt/β-catenin signaling pathway. To do this, we used cholangiocytes and CCA cell line derived from opisthorchiasis-associated CCA. This discovery may offer important insights into the molecular mechanism underlying the infuence of MC-LR on the process of CCA development and hence, possible treatment targeting this signaling pathway. The finding will improve health awareness seeking to limit exposure to MC-LR and hence limiting CCA progression.

## 2 Methods and materials

### 2.1 Main reagents

The material used in this study was Microcystin-leucine arginine (MC-LR) (ALX-350-012-M001; Enzo Life Sciences, NY, USA). To prepare the concentrate solution, 1 mg of MC-LR was suspended in 1000 μL of dimethyl sulfoxide (DMSO), yielding a concentration of 1 mM. To obtain 1, 10, 100, and 1000 nM of MC-LR for each experiment, the concentrate solution was diluted with the completed DMEM medium. These concentrations of MC-LR for treatment groups were selected based on microcystin-polluted surface waters, typically below 100 μg/L (equivalent to approximately 100.5 nM) [29], as well as many previous studies [23,[30], [31], [32]] that have utilized this concentration range.

The β-catenin inhibitor, MSAB (HY-120697; MedChem Express, NJ, USA) was first diluted with DMSO to prepare the stock solution. For the working solution, the inhibitor was further diluted with completed DMEM medium to achieve a 10 μM of MSAB. The inhibitor was treated to the cultured cell 24 h before MC-LR treatment.

### 2.2 Cell culture

The human liver fluke-associated cholangiocarcinoma (CCA) cell line (KKU-213B) and the human immortalized bile duct epithelial cell line (MMNK-1) were used according to a previously described method [33,34]. KKU-213B CCA cell line was established by Sripa et al. [34]. The cells obtained from the Osaka, Japan-based Japanese Collection of Research Bioresources (JCRB) Cell Bank. Cells were cultured in Dulbecco's modified Eagle's medium (DMEM) (11965092, Thermo Scientific, MA, USA) supplemented with 10 % fetal bovine serum (A5256701, Thermo Scientific), and 1 % penicillin-streptomycin (15140122, Thermo Scientific), at 37 °C and 5 % CO_2_. Cells were frequently subcultured using 0.25 % trypsin with EDTA (25200072, Thermo Scientific) once they reached 80 % confluence and then seeded into cell-culture plates for each experiment.

### 2.3 Cell proliferation assay

KKU-213B and MMNK-1 cell lines at a density of 1.5 × 10^3^ cells/well were seeded in 96-well plates (Corning Costar, NY, USA). The cells were treated with various concentrations of MC-LR for 24 h, or exposed to 0.1 % DMSO as a vehicle diluent control on the following day. Cell proliferation analysis was conducted by MTT (3-(4,5-dimethylthiazol-2-yl)-2,5-diphenyltetrazolium bromide) (M6494; Thermo Scientific) assay. To summarize, 4 h of cultivation were allowed after adding 5 mg/mL of MTT solution to each well. After incubation, the solution was aspirated and DMSO was added to dissolve the formazan product. Thermo Scientific's Varioskan LUX microplate reader was used to measure the absorbance at 540 nm. Results were shown as percentage of viable cells and proliferation compared to the corresponding vehicle controls. All assays were performed at least three times.

### 2.4 Phosphatase enzyme activity assay

Cell lines (KKU-213B and MMNK-1) were cultivated on 10 cm Corning Costar cell-culture dishes at 1.5 × 10^6^ cells/dish. The cells were then treated with different concentrations of MC-LR (1, 10, and 100 nM) for 24 h at 37 °C and 5 % CO_2_, or 0.1 % DMSO as a vehicle dilution control. Then, 1 million cells were trypsinized, and phosphatase enzyme was extracted using 1× RIPA buffer (9806; Cell Signaling, MA, USA). 50 μL of supernatant was utilized to detect phosphatase enzyme activity using EnzChek Phosphatase assay kit (E12020; Thermo Scientific) following the manufacturer's guidelines. Afterwards, absorbance of the reaction solution was measured at 358/455 nm by ELISA plate reader. The data were expressed as relative changes in enzyme activity compared to the respective control group. Each assay was conducted in triplicates.

### 2.5 Cell cycle assay

KKU-213B and MMNK-1 cell lines at density of 7.5 × 10^5^ cells/well were seeded in 6-well plates (Corning Costar). They were then treated with different dosages of MC-LR or 0.1 % DMSO as a vehicle dilution control at 37 °C and 5 % CO_2_ for 24 h. The treated cells for the cell cycle experiment were trypsinized, rinsed three times with 1× PBS, fixed with 70 % ethanol, and then stored in ultra-low temperature freezer (−80 °C) for overnight. Cells were then stained with FxCycle propidium iodide (PI)/RNase solution (F10797; Thermo Scientific). BD FACS Canto II (Becton-Dickinson, NJ, USA) was used for the assay of samples, and FlowJo software (also from Becton-Dickinson) was used for data analysis. Three separate experiments were assayed in duplicate.

### 2.6 Wound healing assay

KKU-213B and MMNK-1 cells (9.5 × 10^5^ cells/well) were seeded in 24-well plates (Corning Costar). After the cells had grown to 95 % confluence, a sterile 200-μl plastic pipette tip was used to produce an artificial scratch wound in the well in order to harm the cell monolayer. The cells were exposed to various concentrations of MC-LR and grown at 37 °C and 5 % CO_2_. Images of the scratch wounds were taken 0, 12, and 24 h after injury using an inverted light microscope (Nikon Eclipse Ti2-U, Nikon, Tokyo, Japan). The distance between wound edges was measured using Nikon NIS-Elements software (Nikon). Results were presented as percentage of relative wound closure, analyzed by the formula “Percentage relative wound closure = (scratch wound width at each time point - scratch wound width at time zero) x 100/scratch wound width at time zero”. The gap distance was measured from photographs taken of three randomly selected fields. Experiments were independently conducted triplicate.

### 2.7 Immunofluorescence assay

Immunofluorescence was conducted to localize MC-LR and the OATP1B3 transporter. 2 × 10^4^ cells/well of cell lines were seeded onto 8-well chamber slide (30108, SPL, Gyeonggi-do, ROK) and treated with 100 nM MC-LR or DMSO at 37 °C with 5 % CO_2_ for 24 h. After a certain cultivation time, the cells were fixed with 4 % paraformaldehyde and permeabilized with 0.3 % Triton-X in 1× PBS. After washing twice in 1× PBS, cells were blocked for 1 h in a solution containing 5 % bovine serum albumin in 1× PBS. After being pipetted to the cells, mouse monoclonal anti-microcystin-LR antibody (MC10E7; Enzo Life Sciences) and rabbit polyclonal antibodies against SLCO1B3/OATP1B3 (ab224064; Abcam, Cambridge, UK) were incubated at 4 °C for overnight. After removal of the primary antibodies, fluorescently labeled secondary antibodies, Alexa Fluor 488 goat anti-mouse (A-11029; Thermo Scientific) and Alexa Fluor 594 goat anti-rabbit (A-11012; Thermo Scientific), were added and incubated 2 h at 25 °C. After secondary incubation, samples were stained using Hoechst (Hoechst 33342; Thermo Scientific) to visualize cell nuclei. Fluorescence 2D images were captured by a fluorescence microscopy (Nikon Eclipse Ni-U, Nikon) and a holotomographic microscope (HT-1H Holotomographic Microscopy, Tomocube, Daejeon, ROK) was used for 3D imaging to visualize the deposition of MC-LR in the cells.

### 2.8 Immunoblot analysis

Protease and phosphatase inhibitor (78440, Thermo Scientific)-containing RIPA lysis solution (Cell Signaling) was applied to cell pellets in order to extract proteins. Subsequently, 20 μg of protein were separated via SDS-PAGE and then transferred to PVDF membrane (Cytiva, MA, USA). Following a 5 % BSA block, the membranes were incubated with a rabbit mAb against β-catenin (D10A8; Cell Signaling), β-actin mouse mAb (Ab3280; Abcam), and PP2A rabbit mAb (A6175, Abclonal, MA, USA) overnight at 4 °C. Following washing, membranes were exposed to horseradish peroxidase (HRP)-conjugated secondary antibodies against rabbit (111-035-003; Jackson Immunoresearch, PA, USA) or mouse (7076P2; Cell Signaling) for 2 h. The chemiluminescence reaction was visualized using ECL reagent (WBLUF0100; Merck, Darmstadt, Germany), and bands were detected using Amersham ImageQuant 800 (GE Healthcare, IL, USA). The bands intensity of protein was assessed utilizing ImageJ software (National Institutes of Health (NIH), MD, USA). The data come from three independent experiments.

### 2.9 qRT-PCR analysis

RNA was extracted from cell lines using RNA extraction kit (12183018A, Thermo Scientific). cDNA was constructed using the cDNA synthesis kit (K1622; Thermo Scientific) according to the manufacturer's guideline. To detect Wnt/β-catenin-related genes, including *Axin1*, *TCF-7*, *APC*, *c-Jun*, and *Cyclin D1*, the primer pairs were used as previously described. The specific primer pair for *Axin 1* was as follows: F; 5′ GACCTGGGGTATGAGCCTGA 3′, R; 5′ GGCTTATCCCATCTTGGTCATC 3′, and F; 5′ TTGATGCTAGGTTCTGGTGTACC 3′ R; 5′ CCTTGGACTCTGCTTGTGTC 3′ for the *TCF-7* gene [35] and F; 5′ CCTCATCCAGCTTTTACATGGC 3′ and R; 5′ GCCCGAGCCTCTTTACTGC 3′ for the *APC* gene [36] and F; 5′ GTCCTTCTTCTCTTGCGTGG 3′ R; 5′ GGAGACAAGTGGCAGAGTCC 3′ for the *c-Jun* gene [37] and F; 5′ AGATGAAGGAGACCATCCCCC 3′ and R; 5′ CCACTTGAGCTTGTTCACCA 3′ for the *Cyclin D1* gene [38]. The qRT-PCR reaction included SYBR Green I Master (Roche, Mannheim, Germany) and 2 μg of cDNA template. The PCR condition in a LightCycler 480 II system (Roche) were as follows: annealing at 95 °C for 5 min; 40 cycles of 95 °C for 10 s, 58 °C for *Axin1* and *c-Jun* genes, 61 °C for *TCF-7* and *APC* genes, and 62 °C for *Cyclin D1* genes. Using the β-actin as a housekeeping gene, the 2^‾ΔΔCT^ technique [39] was employed to assess the proportions of gene expression compared to controls. Relative gene expression level was measured independently in three different samples.

### 2.10 Immunohistochemical studies

Tissue samples from 50 CCA patients were dissected into 5 μm sections and treated with xylene to remove paraffin, then gradually rehydrated with 100 %, 95 %, and 70 % ethanol. To recover antigen, sections were autoclaved in citrate buffer at 110 °C for 10 min. Immunostaining was conducted by incubating slides at 4 °C for overnight with a β-catenin rabbit mAb (A19657; Abclonal). Goat anti-rabbit conjugated to horseradish peroxidase (HRP) (111-035-003; Jackson Immunoresearch) was applied to the slides following incubation. Immunoreactivity was visualized by addition of DAB solution (3′,3′-diaminobenzidine) (ES005, Merck). Then, slide was counterstained with Mayer's hematoxylin for 2 min, followed by rinsing with distilled water and dehydration using 70 %, 95 %, absolute ethanol, and xylene. Ten representatives and randomly selected areas of each slide were examined from the Aperio ImageScope software (Leica biosystems, IL, USA) using 40× magnification. The relative intensity was assessed using ImageJ software (NIH). In addition, to detect MC-LR in CCA tissue, the MC-LR mouse mAb (MC10E7; Enzo Life Sciences) was also used according to previously described method [30].

### 2.11 ELISA assay

We used the microcystins (MCs) ELISA kit (502000; Cayman Chemical, MI, USA) in accordance with the manufacturer's instructions to measure the concentration of MC-LR in the serum of both CCA patients (n = 20) and healthy individuals (n = 10), in order to confirm the existence of MC-LR in CCA patients. In brief, 50 μL of both samples and microcystin (MC) standard solutions were carefully added to the designated test wells. Subsequently, microcystin-HRP tracer and microcystin ELISA monoclonal antibodies were then added to the wells. The subsequent phase comprised a precisely controlled incubation period of 2 h at 25 °C. After this incubation, an ELISA reader (Varioskan LUX, Thermo Scientific) was assayed to evaluate the absorbance at 450 nm. In addition, serum spiked with MCs was used as positive control.

### 2.12 Statistical analysis

Results from triplicates independently experiments are presented as mean ± standard deviation (SD). To compare the significant difference means between two groups, the student's t-test was used. Tukey's post hoc analysis was conducted after one-way analysis of variance (one-way ANOVA) analysis to assess the significant difference means among experimental groups. GraphPad Prism version 9.1 (GraphPad Software, MA, USA) and SPSS 26 software (SPSS Inc, IL, USA) were used for statistical analysis. A significance level was determined for P values less than 0.05.

## 3 Results

### 3.1 Uptake and accumulation of MC-LR lead to greater cell proliferation and migration

**Fig. 1:**
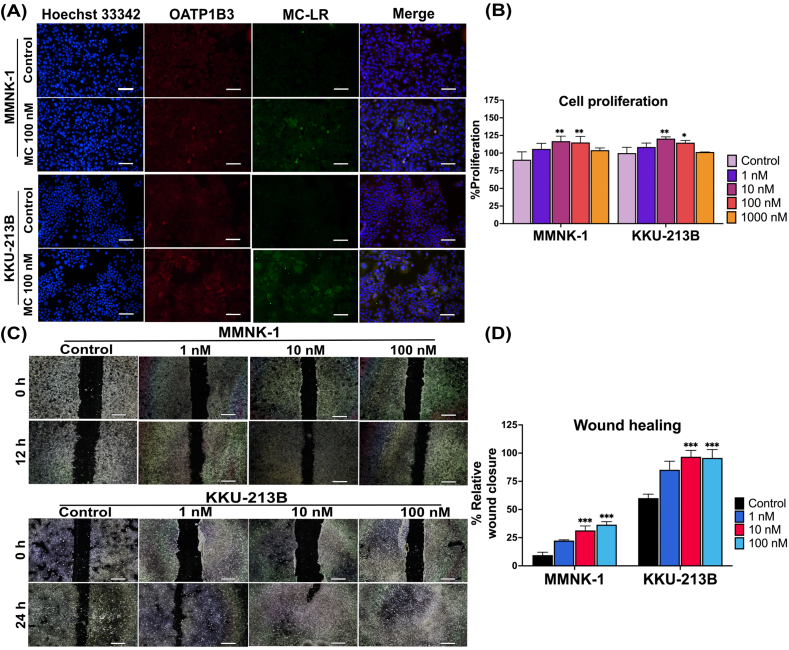
Uptake of MC-LR by cholangiocytes and CCA cells increases cell proliferation and migration. (A) Colocalization of Alexa Flour 488-labeled MC-LR (green) and Alexa Fluor 594-labeled OATP1B3 (red) were investigated using a fluorescence microscope (scale = 100 μm). (B) MC-LR induces proliferation of cholangiocytes and CCA cells as measured by the MTT assay. (C) Representative light microscopy images of the wound healing assay show the effect of MC-LR on migration by cholangiocytes and CCA cells (scale = 200 μm). (D) Summary bar graphs show the percentage of wound closure at the indicated time points during the scratch wound experiment. All experiments were performed at least in triplicate. Graphs are presented as mean ± SD. Statistical analysis was performed using one-way ANOVA, followed by Tukey's test (*P < 0.05, **P < 0.01, ***P < 0.001 vs control group). (For interpretation of the references to color in this figure legend, the reader is referred to the Web version of this article.)

The expression of the OATP1B3 transporter and the cellular accumulation of MC-LR were examined using the immunofluorescence. The findings demonstrated that MMNK-1 and KKU-213B cells allowed the uptake of MC-LR, and MC-LR can accumulate in the cells (Fig. 1A). In addition, holotomography methods verified the accumulation of MC-LR within the cell (Supplementary videos 1–4).

The effect of MC-LR on MMNK-1 and KKU-213B cells was examined using the MTT assay (Fig. 1B). The data demonstrated a significant increase in the proliferation of KKU-213B cells at concentrations of 10 nM (P < 0.01) and 100 nM (P < 0.05) in comparison to the vehicle control, as well as in MMNK-1 cells with 10 nM and 100 nM of MC-LR (P < 0.01). In addition, the wound healing assay showed that 10 nM and 100 nM of MC-LR significantly activated the migration of MMNK-1 cells at 12 h and KKU-213B cells at 24 h compared to controls (P < 0.001, Fig. 1C and D). These findings indicate that both cell lines have the capability to uptake MC-LR, that it affects cellular processes and contributes to enhanced cellular proliferation and migration.

### 3.2 MC-LR causes the cell cycle to shift from G0/G1 to S phase

**Fig. 2:**
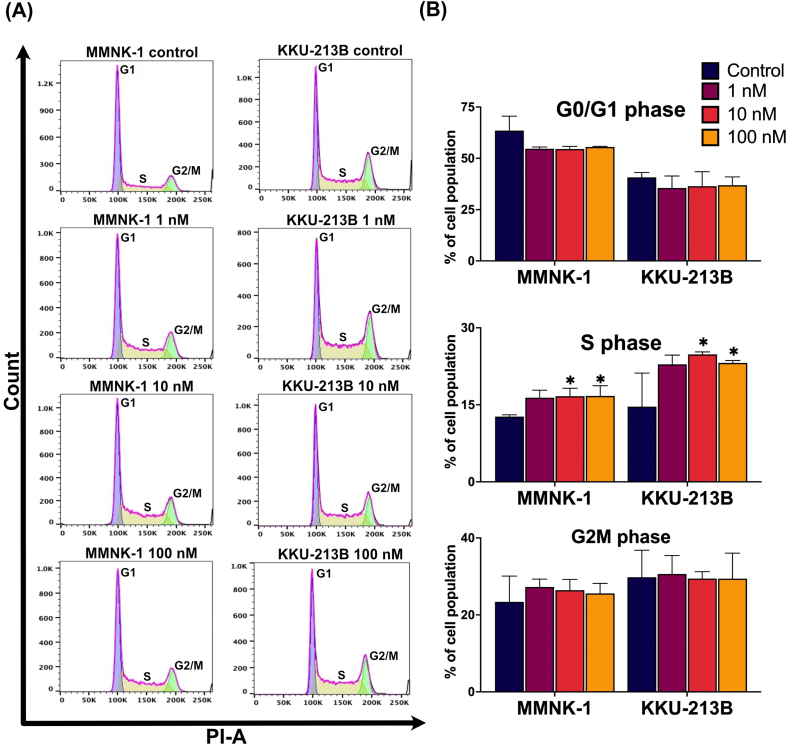
MC-LR induces cell cycle progression in MMNK-1 and KKU-213B cell lines. (A) The percentage of cells in each phase of the cell cycle is shown graphically. (B) Results show a significant increase in the proportion of cells in the S phase compared to the control group. The experiment was performed in triplicate (*P < 0.05 vs control group). Graphs are presented as mean ± SD. Statistical analysis was performed using one-way ANOVA, followed by Tukey's test (*P < 0.05 vs control group).

We used flow cytometry to verify the impact of MC-LR on the cell cycle of MMNK-1 and KKU-213B cells. Compared to the vehicle control, the data showed a significantly higher percentage of cells in S phase for both cell lines following 10 nM and 100 nM MC-LR treatment (P < 0.05, Fig. 2A and B). According to these studies, MC-LR stimulates the G0/G1 phase to S phase transition, which has an impact on the cell cycle.

### 3.3 MC-LR inhibits phosphatase enzyme and activates Wnt/β-catenin signaling

**Fig. 3:**
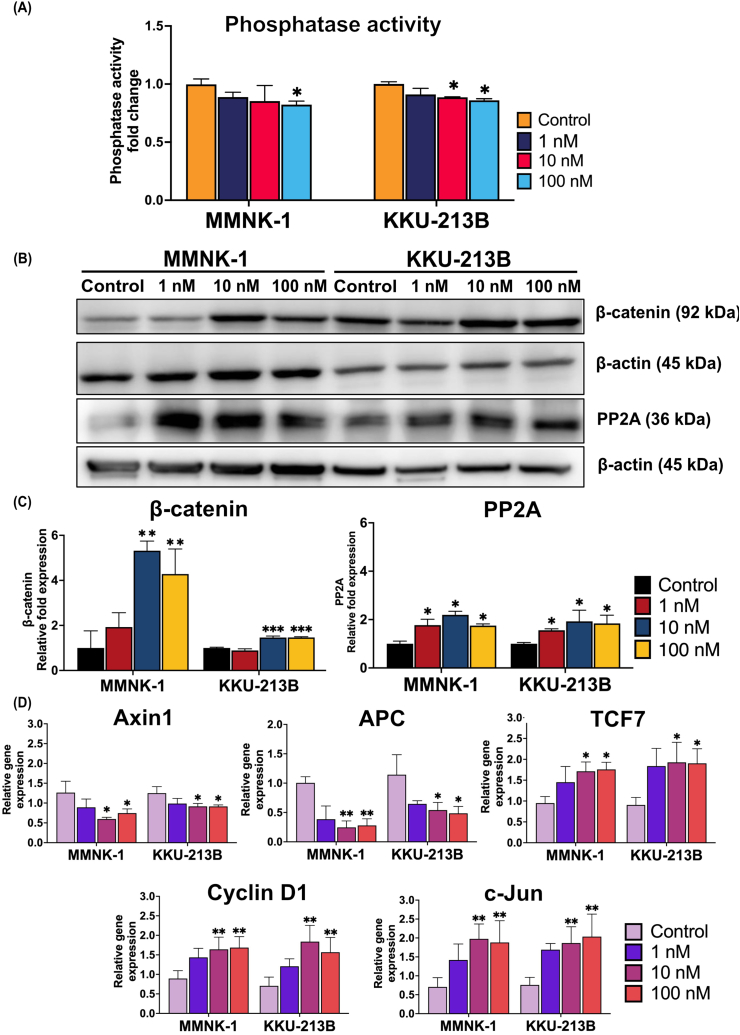
MC-LR reduces phosphatase enzyme activity, induces expression of β-catenin and up-regulates β-catenin target genes in MMNK-1 and KKU-213B cell lines. (A) MC-LR reduces phosphatase enzyme activity in cholangiocyte and CCA cell lines. (B) The expression of β-catenin and PP2A in the cell lines was examined by western blotting. (C) The ratio of the proteins were determined by densitometry and normalized to β-actin as an internal control. Data are presented as mean ± SD. (D). Gene expression levels of Wnt/β-catenin signaling molecules were detected by qRT-PCR and normalized to the β-actin gene. Graphs are presented as mean ± SD. All experiments were performed in triplicate. Statistical analysis was performed using one-way ANOVA, followed by Tukey's test (*P < 0.05, **P < 0.01, ***P < 0.001 vs control group).

Using a phosphatase detection kit, we investigated how MC-LR affects the cellular phosphatase activity. Our results demonstrated a significant decrease in phosphatase activity in MMNK-1 cells treated with 100 nM MC-LR, and in KKU-213B cells treated with 10 nM and 100 nM MC-LR (P < 0.05, Fig. 3A). Immunoblot analysis revealed elevated levels of β-catenin and PP2A proteins in both cell lines after treated with 1, 10, and 100 nM of MC-LR (Fig. 3B). In the MC-LR treatment groups, the expression of β-catenin protein was considerably higher at doses of 10 nM and 100 nM compared to the vehicle control group (P < 0.01 for MMNK-1 and P < 0.001 for KKU-213B, Fig. 3C), while expression of PP2A significantly upregulated after three doses treatment (P < 0.05, Fig. 3C). Additionally, the expression of destruction-complex genes (Fig. 3D), including *Axin1* (P < 0.05) and *APC* (P < 0.01 for MMNK-1 and P < 0.05 for KKU-213B), significantly decreased when treated with 10 nM and 100 nM MC-LR. Conversely, the same treatment groups exhibited significantly overexpression of Wnt/β-catenin target genes (Fig. 3D), such as *TCF-7* (P < 0.05), *Cyclin D1* and *c-Jun* (P < 0.01). These data suggest that MC-LR can enter cells and stimulate the Wnt/β-catenin signaling pathway, which in turn promotes cell proliferation.

### 3.4 Inhibition of β-catenin induces downregulation of Wnt/β-catenin target genes after MC-LR exposure

**Fig. 4:**
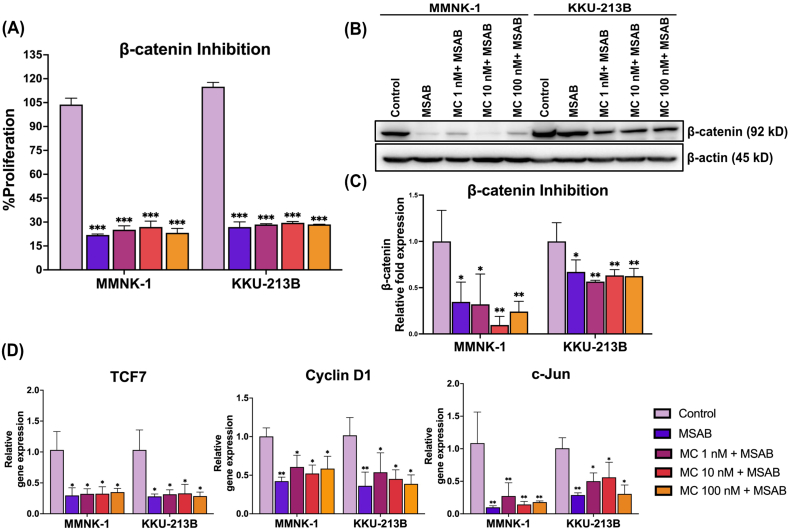
Downregulation of Wnt/β-catenin target genes and reduction of cell proliferation are observed when the accumulation of β-catenin is inhibited by MSAB. (A) Blocking the β-catenin signaling pathway with the inhibitor, MSAB, leads to a decrease in proliferation of cholangiocytes and CCA cells. Cell proliferation was determined using the MTT assay after treatment with MSAB. (B) The expression of β-catenin was detected by western blotting. (C) The ratio of the proteins was determined by densitometry and normalized to β-actin as an internal control. Data are presented as mean ± SD. (D) The expression levels of Wnt/β-catenin target genes were determined by qRT-PCR and normalized to β-actin as a housekeeping gene. All data are presented as mean ± SD. Experiments were performed at least in triplicate. Statistical analysis was performed using one-way ANOVA, followed by Tukey's test (*P < 0.05, **P < 0.01, ***P < 0.001 vs control group).

An inhibitor targeting β-catenin (MSAB) was used to inhibit β-catenin in MMNK-1 and KKU-213B cells, and cell proliferation was then assessed to validate the effect of the Wnt/β-catenin pathway on regulating the proliferation of cholangiocytes and CCA cells. Compared to the vehicle control (without MSAB treatment), the combination of MSAB and MC-LR (at 1 nM, 10 nM, and 100 nM) significantly reduced cell proliferation in both cell lines (P < 0.001, Fig. 4A). The expression of β-catenin protein was significantly reduced by MSAB alone (P < 0.05), and by MC-LR 10 nM + MSAB (P < 0.01) or MC-LR 100 nM + MSAB (P < 0.01) (Fig. 4B and C). Subsequently, downregulation of β-catenin target genes was also observed in both cell lines, including *TCF7* (P < 0.05, all treated groups), *Cyclin D1* (P < 0.01, MSAB treatment or P < 0.05, MC-LR + MSAB), and *c-Jun* (MMNK-1: P < 0.01, all treated groups; KKU-213B: P < 0.01, MSAB treatment or P < 0.05, MC-LR + MSAB) (Fig. 4D). These findings suggest that inhibition of this pathway results in a reduction of proliferation and migration even in the presence of MC-LR.

### 3.5 Overexpression of β-catenin in the cancerous tissue of CCA patients

**Fig. 5:**
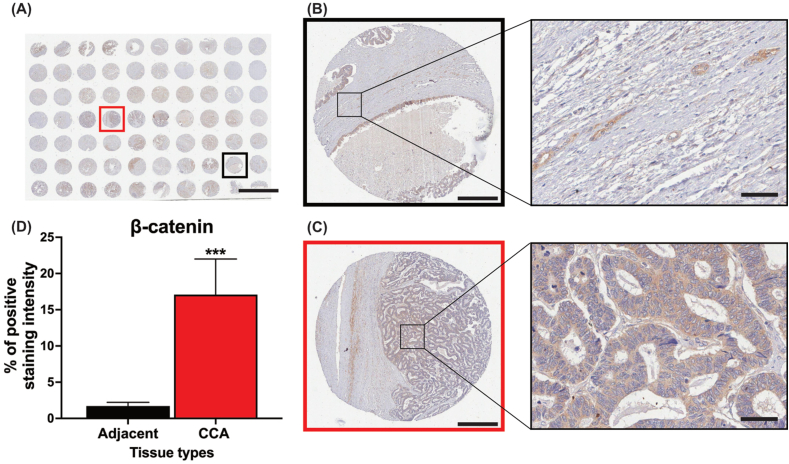
The overexpression of β-catenin in CCA tissue. (A) The tissue array was stained with anti-β-catenin and captured at 1× magnification (scale = 5 mm). (B) The expression levels of β-catenin were assessed in non-tumor adjacent tissues (black box) using immunohistochemical techniques (scale = 50 μm). (C) The expression levels of β-catenin were assessed in CCA tissues (red box) using immunohistochemical techniques (scale = 50 μm). (C) The positive staining (brown color) intensity of β-catenin in the tissues was quantified using ImageJ software. The graph represents the mean ± SD of 10 representative areas per sample. Statistical analysis was performed using Student's t-test (***P < 0.001 vs adjacent area). (For interpretation of the references to color in this figure legend, the reader is referred to the Web version of this article.)

An immunohistochemical technique was conducted to examine the β-catenin expression in CCA tissues (Fig. 5A). Notably, the CCA area showed both strong and moderate expression of β-catenin. On the other hand, β-catenin protein's immunoreactivity in adjacent tissues was much lower than in tumorous tissues (P < 0.001, 5B-D). This result suggests that β-catenin might be involved in cholangiocarcinogenesis.

### 3.6 Identification of MC-LR in clinical specimens

We conducted both immunohistochemical (IHC) and ELISA assays to determine the occurrence of microcystins (MCs) in the tissues and serum of CCA patients. Contrary to our expectations, our study revealed that there was no statistically notable contrast in MCs levels among samples collected from individuals without health issues (0.0434 ± 0.0159 ng/mL) and those from CCA patients (0.0161 ± 0.0139 ng/mL). Also, our efforts to localize MCs in CCA tissues were inconclusive and therefore these results are not available for presentation (data not shown).

## 4 Discussion and conclusion

Microcystins (MCs) represent the most frequently identified cyanotoxins in freshwater bodies worldwide [40,41], including in northeastern Thailand, where opisthorchiasis-associated CCA is most prevalent [4]. Although microcystin-leucine-arginine (MC-LR) is the MC with the highest potential for carcinogenicity [8,9]; its effect on cholangiocarcinogenesis is unclear. We have demonstrated here for the first time that MC-LR promotes cell cycle progression through the Wnt/β-catenin pathway and CCA progression, which has also been connected to the liver fluke infection and CCA development [17]. Expression of β-catenin in CCA is higher than in adjacent tissues, indicating its significant role in cholangiocarcinogenesis.

The organic anion polypeptide transporters (OATPs), which mediate the transport of drugs and various xenobiotics into the cell, are involved in cellular uptake of MC-LR [42]. OATP1B3 exhibits a higher capacity for MC-LR uptake than other OATPs [43,44] in numerous cancers, such as liver cell carcinoma, pancreatic cancer, and colon cancer [[45], [46], [47]]. Our study revealed that both MMNK-1 and KKU-213B cells express OATP1B3, which enables them to efficiently take up MC-LR into cells.

The concentrations of MC-LR employed in our study are comparable to those found in microcystin-polluted surface waters, typically below 100 μg/L (equivalent to approximately 100.5 nM) [29]. MC-LR is recognized for its dual impact on cells, eliciting cell death at high concentrations (>10 μM) [48,49] and promoting cell proliferation at lower concentrations (<500 nM) [23,30,32]. In our study, we found that 10 nM and 100 nM MC-LR stimulated cell division and migration in CCA and cholangiocytes cells at certain concentrations (10 nM and 100 nM) but not at lower (1 nM) or higher (1000 nM) concentrations. These results suggest that high doses (>1000 nM) of MC-LR could induce cellular toxic effects such as apoptosis and necrosis [[48], [49], [50]], while low doses (1 nM in our study and 0.5 and 5 nM in a previous study [30]) failed to promote intrahepatic cholangiocarcinoma cell proliferation [30]. Exposure to low doses of MC-LR (25–100 nM) have been reported to accelerate cancer migration and metastasis [[51], [52], [53]], implying that it might be essential for the development of cancer by promoting the overexpression of proto-oncogenes including *c-Jun*, *c-Myc*, and *c-Fos* [[52], [53], [54], [55]]. Several studies indicate that MC-LR can potentially promote the proliferation of cancer, including CCA. In our study, the proportion of cells in S phase increased after treatment with MC-LR. This observation is in line with recent studies using intrahepatic biliary epithelial cells [32] and fibroblasts cells [31], indicating that MC-LR has the potential to shift cells from G0/G1 phase to S phase, thus promoting cell proliferation. The increased expression of *Cyclin D1* supports MC-LR's impact on the cell cycle, which can potentially impact on the proliferation of cholangiocytes and CCA cells.

The mechanism through which MC-LR induces cancer involves its inhibition of PP1/PP2A [56], molecules within the Wnt/β-catenin pathway that contribute to the activation of the β-catenin destruction complex [57]. Our result showed that the activity of the phosphatase enzyme was lower under MC-LR-treated conditions than under the vehicle control. Furthermore, the groups treated with MC-LR showed increased PP2A protein expression. This outcome is in line with earlier research that shown how MC-LR can interrupt the activity of phosphatase enzyme, which in turn promotes the production of PP2A [58,59]. This result suggests that MC-LR has the ability to impede the activity of phosphatase enzymes in the cells and consequently exert an influence on cell signaling cascades including Wnt/β-catenin pathway. Axin1 and adenomatous polyposis coli (APC), the fundamental elements of the β-catenin destruction complex, are required to regulate carcinogenesis and cancer progression [60,61]. Consistent with this, our evidence revealed that administration of MC-LR to both cell lines resulted in downregulation of gene expression for *Axin1* and *APC*. Disrupting the β-catenin destruction complex causes overabundance of β-catenin, resulting in dysregulation of the Wnt/β-catenin pathway. This is a frequent feature in many aggressive malignancies, such as liver cell carcinoma, breast cancer, and colon cancer [62]. Furthermore, our results (Fig. 3B–D) align with a previous report by Pan C. et al. which demonstrated that exposure to MC-LR could promote benign prostatic hyperplasia via β-catenin-mediated pathways *in vivo* [63]. β-catenin exerts its function by binding with *TCF* and activates target genes, including *c-Jun* and *Cyclin D1* [27,64]. Following MC-LR treatment, *TCF* and *Cyclin D1* gene expression was upregulated in both cell lines (Fig. 3D). These findings align with prior research demonstrating that β-catenin and its downstream molecules, including Cyclin D1, are upregulated in colorectal cancer cells treated with MC-LR [23]. Increased levels of Cyclin D1 and β-catenin proteins have been linked to unfavorable survival outcomes in cancer patients, and Cyclin D1 is known to play crucial roles in cancer development and progression [65,66]. Moreover, our findings indicated an increase in *c-Jun* expression following exposure to MC-LR, aligning with prior research suggesting that MCs can induce *c-Jun* transcription [67]. Significantly, *Cyclin D1*, *c-Jun*, and *TCF* are associated with both cell proliferation and tumor growth. This discovery was verified using MSAB, a specific inhibitor of the Wnt/β-catenin pathway (Fig. 4), consistent with a previous publication [68].

To further substantiate our findings, β-catenin in CCA biopsy tissues was stained by immunohistochemical technique. The results indicated that the malignant area had a significant higher expression of β-catenin compared to the adjacent tissue. This is consistent with previous research linking dysregulation of the Wnt/β-catenin signaling pathway to CCA associated with liver fluke infections [17]. Overall, MC-LR interrupt the β-catenin destruction complex, resulting in overexpression of β-catenin in the cytoplasm. This implies that β-catenin might be essential for the carcinogenesis of cancer [28], especially in cholangiocarcinogenesis processes. Therefore, the Wnt/β-catenin signaling pathway could be a suitable target for both treatment and cancer prevention.

MCs are known to be hepatotoxic and carcinogenic [9]. Chronic exposure may increase risk of liver cell carcinoma [69]. Despite the lack of solid evidence, a number of lines of investigation have indicated a possible association between MC-LR exposure and CCA. The toxin was discovered inside the intrahepatic bile duct of an animal model that had received chronic MC-LR exposure, eventually leading to bile-duct hyperplasia [32]. After receiving MC-LR treatment for 24 h, an *in vitro* study also found MC-LR in human intrahepatic bile duct epithelial cells [30], similar to human hepatocytes [70]. The induction of inflammatory and proinflammatory cytokines, along with chemokines, can be induced by MC-LR exposure, resulting in the infiltration of inflammatory cells [71]. Hence, MC-LR may also promote chronic inflammation that contributes to the development of cancer, similar to liver fluke-mediated CCA [25,72,73]. The level of MC-LR is a factor in the poor prognosis of CCA patients in both animal models and *in vitro* [30]. Taken together, this evidence supports the hypothesis that MC-LR may reach not only hepatocytes but also biliary epithelial cells, thereby contributing to development and progression of CCA.

In addition to the *in vitro* investigation, we measured MC-LR levels in sera from CCA patients and found no significant difference between CCA and healthy volunteers. We could not detect MC-LR in CCA tissues. This could be explained by the fact that MCs have a short half-life, typically 3–10 weeks [74], lower uptake rate in liver tissue (1.1 % of exposure) [75], or low concentrations in water bodies or the environment in northeastern Thailand (0.913 μg/L) [4]. However, overexpression of β-catenin was seen in the MC-LR treated cells as well as in CCA tissues, suggesting a role for MC-LR in this upregulation. Based on a two-stage carcinogenesis model [76], MC-LR could be considered a tumor promoter where repeated and prolonged exposure is necessary for cancer development. Despite our discovery that lower doses of MC-LR, specifically at 10 nM and 100 nM, were capable of enhancing the migration and proliferation of CCA and cholangiocytes cells *in vitro*, further studies, especially in animal models of CCA, are required to confirm whether these doses are appropriate and whether they could enhance the development and progression of CCA.

**Fig. 6:**
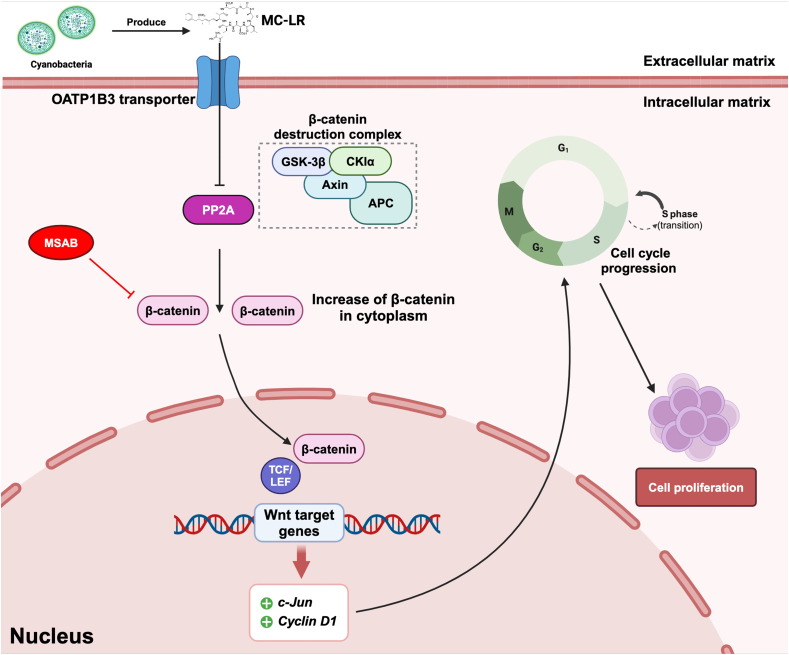
The postulated mechanism by which MC-LR promotes proliferation of cholangiocytes and cholangiocarcinoma (CCA) cells. This is as follows: MC-LR disrupts the β-catenin destruction complex through the inhibition of PP2A, leading to the diffuse of β-catenin into the cell nucleus. Within the nucleus, β-catenin activates Wnt/β-catenin target genes, including *c-Jun* and *Cyclin D1*, thereby contributing to the proliferation of cholangiocytes and CCA cells. The figure has been created using BioRender (www.biorender.com).

**In conclusion,** this study offers valuable insights into how MC-LR impacts the proliferation and progression of CCA. Our study provides strong evidence that MC-LR stimulates the overgrowth of cholangiocytes and CCA cells, partially through Wnt/β-catenin signaling pathway. A summary model of this research is shown in Fig. 6. This knowledge could be important for the creation of focused treatment plans that slow the progression of CCA. The discovery of MC-LR as a possible promoter of CCA growth emphasizes the necessity for additional research in this field. It will be important to reduce human exposure to MCs in the environment to limit the development and progression of CCA.

## Ethics statement

The study protocol was based on the Declaration of Helsinki and the ICH Good Clinical Practice Guidelines. This study was approved by the Khon Kaen University Human Research Ethics Committee under reference number HE 641416 for cell lines, reference number HE 641489 and HE 661518 for application in clinical study.

## Funding

The funding for this study was provided by the 10.13039/501100004071Khon Kaen University Research Fund (Grant no. KKU620009004) and Basic Research Fund of 10.13039/501100021795Khon Kaen University under the Cholangiocarcinoma Research Institute (CARI-BRF64-5). Suppakrit Kongsintaweesuk acknowledges the Centre for Research and Development of Medical Diagnostic Laboratories (CMDL), Faculty of Associated Medical Sciences, Khon Kaen University, Thailand. The funders were not involved in the study's design, data collection and analysis, publication decision, or manuscript preparation.

## CRediT authorship contribution statement

**Suppakrit Kongsintaweesuk:** Writing – original draft, Visualization, Validation, Methodology, Investigation, Formal analysis, Conceptualization. **Sirinapha Klungsaeng:** Writing – review & editing, Validation, Methodology, Formal analysis. **Kitti Intuyod:** Writing – review & editing, Validation, Resources, Conceptualization. **Anchalee Techasen:** Writing – review & editing, Validation. **Chawalit Pairojkul:** Validation, Resources. **Vor Luvira:** Validation, Resources. **Somchai Pinlaor:** Writing – review & editing, Validation, Methodology. **Porntip Pinlaor:** Writing – review & editing, Writing – original draft, Validation, Supervision, Project administration, Methodology, Investigation, Funding acquisition, Formal analysis, Conceptualization.

## Declaration of competing interest

The authors declare that they have no known competing financial interests or personal relationships that could have appeared to influence the work reported in this paper.
