# Supplementary material for: Microcystin-leucine arginine induces the proliferation of cholangiocytes and cholangiocarcinoma cells through the activation of the Wnt/β-catenin signaling pathway
Source: Heliyon. 2024 Apr 26;10(9):e30104. doi: 10.1016/j.heliyon.2024.e30104 (PMC11076882; doi:10.1016/j.heliyon.2024.e30104)
Supplement: Multimedia component 1 [file mmc1.pdf]

Full-length blot

β-catenin 92 kDa:

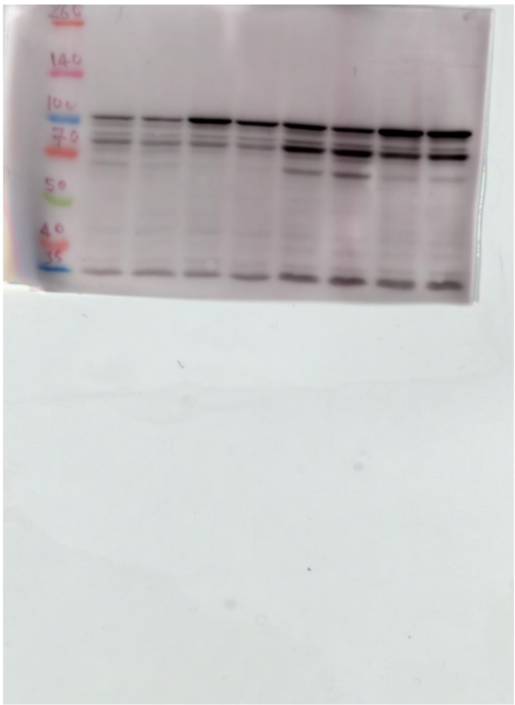

Left Right

Fig. S1 The full-length blot illustrates the protein bands of β-catenin in MMNK-1 and KKU-213B cells treated with various concentration of MC-LR.

From left to right:

lane 1; marker

lanes 2-5; for MMNK-1 treated with 0.1% DMSO as control, MC-LR 1nM, MC-LR 10 nM and MC-LR 100 nM, respectively.

Lanes 6-9 for KKU-213B treated with 0.1% DMSO as control, MC-LR 1nM, MC-LR 10 nM and MC-LR 100 nM, respectively.

This original blot was used as a representative band for visualization of β-catenin protein in Figure 3 in the main manuscript.

Beta-actin 45 kDa:

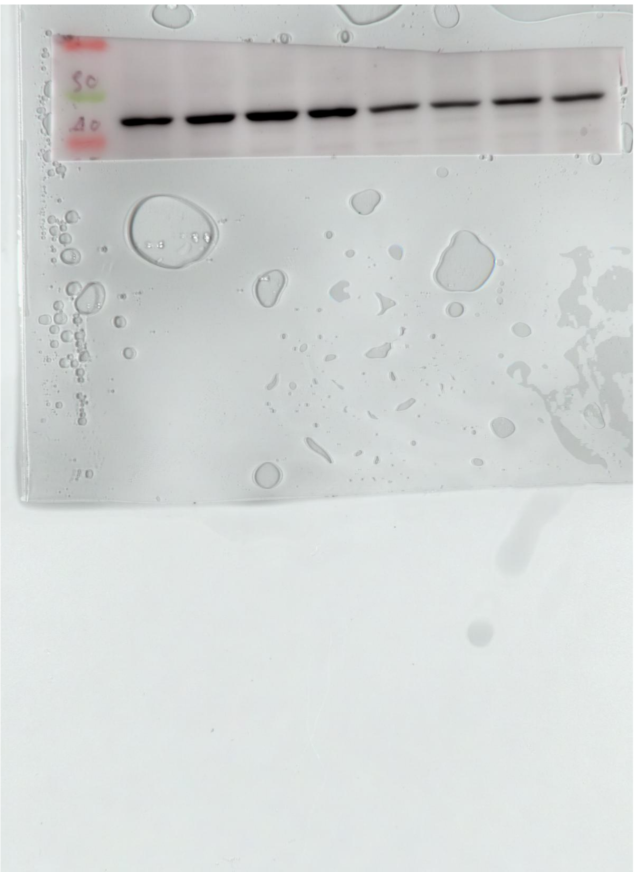

Left Right

Fig. S2 The full-length blot illustrates the protein bands of β-actin in MMNK-1 and KKU-213B cells treated with various concentration of MC-LR.

From left to right:

lane 1; marker

lanes 2-5; for MMNK-1 treated with 0.1% DMSO as control, MC-LR 1nM, MC-LR 10 nM and MC-LR 100, respectively.

Lanes 6-9 for KKU-213B treated with 0.1% DMSO as control, MC-LR 1nM, MC-LR 10 nM and MC-LR 100, respectively.

This original blot was used as a representative band for visualization of β-actin protein in Figure 3 in the main manuscript.

PP2A 36 kDa:

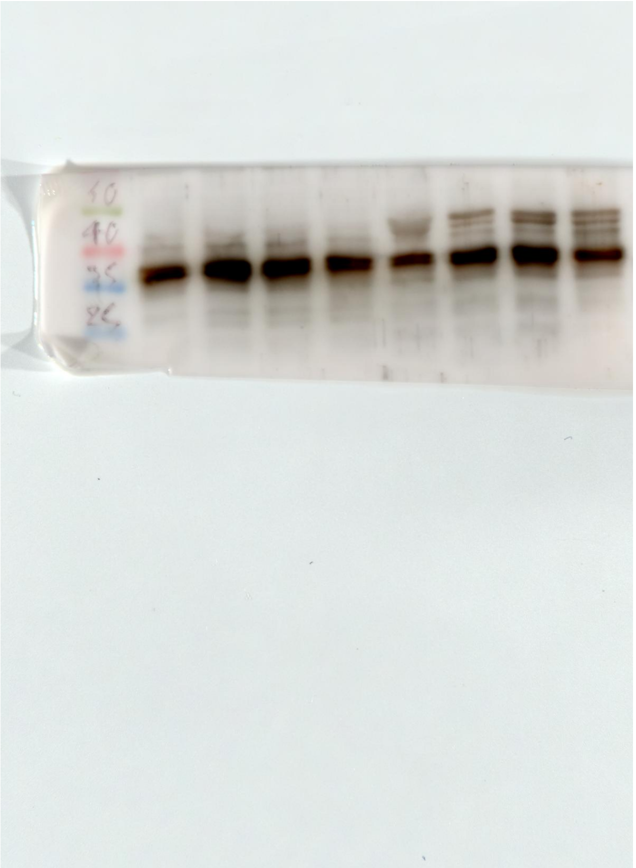

Left Right

Fig. S3 The full-length blot illustrates the protein bands of PP2A in MMNK-1 and KKU-213B cells treated with various concentration of MC-LR.

From left to right:

lane 1; marker

lanes 2-5; for MMNK-1 treated with 0.1% DMSO as control, MC-LR 1nM, MC-LR 10 nM and MC-LR 100, respectively.

Lanes 6-9 for KKU-213B treated with 0.1% DMSO as control, MC-LR 1nM, MC-LR 10 nM and MC-LR 100, respectively.

This original blot was used as a representative band for visualization of PP2A protein in Figure 3 in the main manuscript.

β-actin 45 kDa:

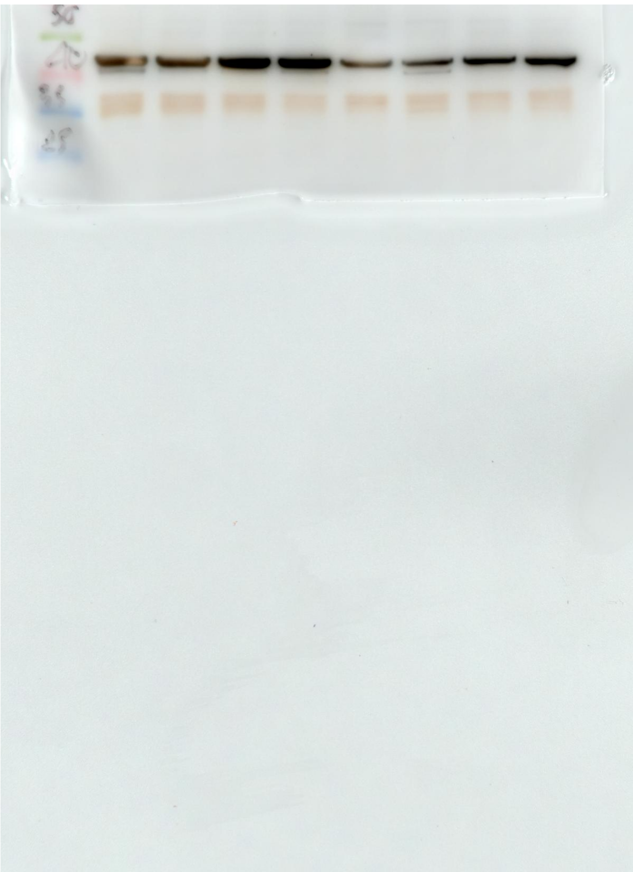

Left Right

Fig. S4 The full-length blot illustrates the protein bands of β-actin in MMNK-1 and KKU-213B cells treated with various concentration of MC-LR.

From left to right:

lane 1; marker

lanes 2-5; for MMNK-1 treated with 0.1% DMSO as control, MC-LR 1nM, MC-LR 10 nM and MC-LR 100, respectively.

Lanes 6-9 for KKU-213B treated with 0.1% DMSO as control, MC-LR 1nM, MC-LR 10 nM and MC-LR 100, respectively.

This original blot was used as a representative band for visualization of β-actin protein in Figure 3 in the main manuscript.

β-catenin 92 kDa:

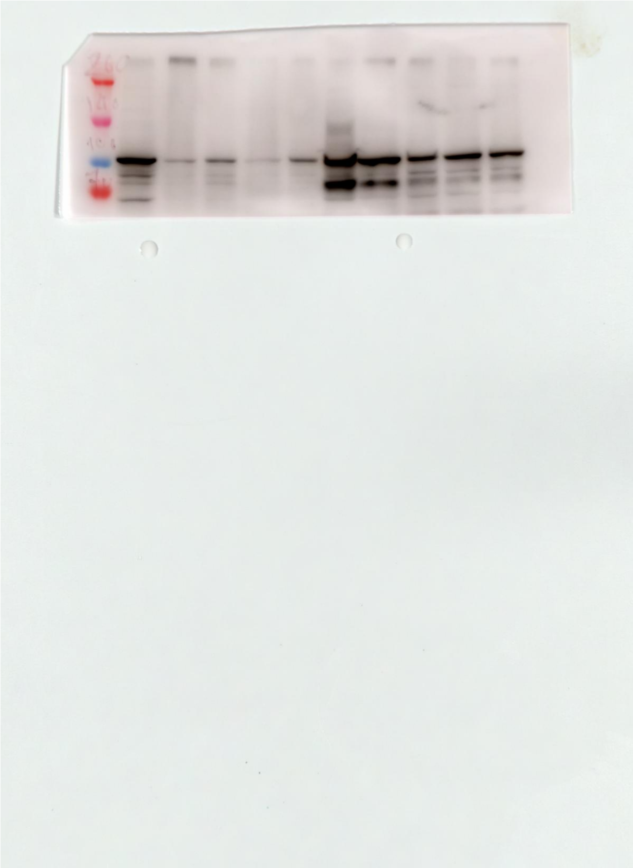

Left Right

Fig. S5 The full-length blot illustrates the protein bands of β-catenin in MMNK-1 and KKU-213B cells treated with MSAB and various concentration of MC-LR.

From left to right:

lane 1; marker

lanes 2-6; for MMNK-1 treated with 0.1% DMSO as control, MSAB, MC-LR 1nM+MSAB, MC-LR 10 nM+MSAB and MC-LR 100 nM+MSAB, respectively.

Lanes 7-11 for KKU-213B treated with 0.1% DMSO as control, MSAB, MC-LR 1nM+MSAB, MC-LR 10 nM+MSAB and MC-LR 100 nM+MSAB, respectively.

This original blot was used as a representative band for visualization of β-catenin protein in Figure 4 in the main manuscript.

Beta-actin 45 kDa:

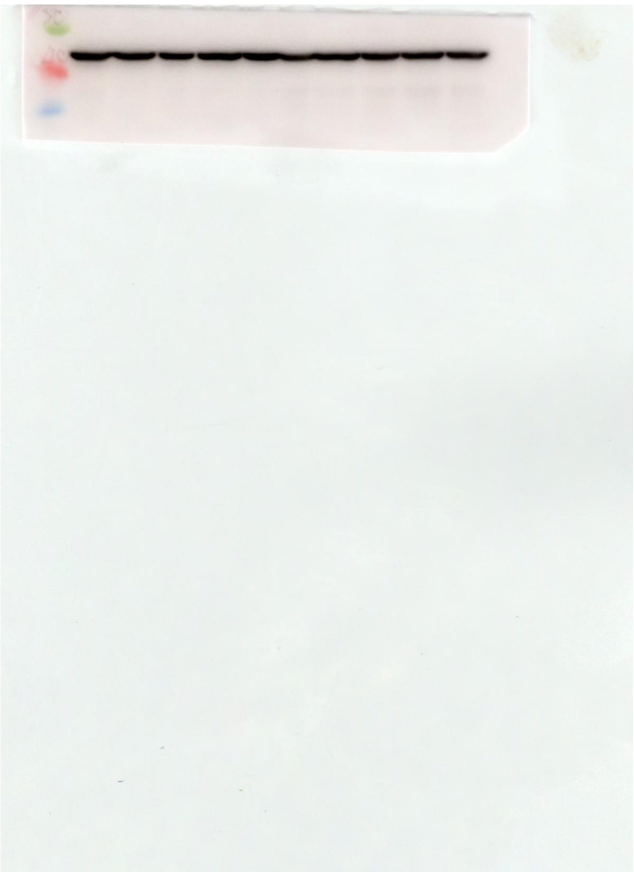

Left Right

Fig. S6 The full-length blot illustrates the protein bands of β-actin in MMNK-1 and KKU-213B cells treated with MSAB and various concentration of MC-LR.

From left to right:

lane 1; marker

lanes 2-6; for MMNK-1 treated with 0.1% DMSO as control, MSAB, MC-LR 1nM+MSAB, MC-LR 10 nM+MSAB and MC-LR 100 nM+MSAB, respectively.

Lanes 7-11 for KKU-213B treated with 0.1% DMSO as control, MSAB, MC-LR 1nM+MSAB, MC-LR 10 nM+MSAB and MC-LR 100 nM+MSAB, respectively.

This original blot was used as a representative band for visualization of β-actin protein in Figure 4 in the main manuscript.
